# Trends in the Prevalence of Macrosomia Among Live Births in Greece: A Nationwide Analysis, 1980-2023

**DOI:** 10.7759/cureus.110245

**Published:** 2026-06-04

**Authors:** Nikolaos Vlachadis, Chrysi Christodoulaki, Rafaela Panagopoulou, Ioanna Petrakou, Nikolaos Machairiotis, Konstantinos Louis, Dimos Sioutis, Maria Zoi Bourou, Sofoklis Stavros, Periklis Panagopoulos

**Affiliations:** 1 Third Department of Obstetrics and Gynecology, National and Kapodistrian University of Athens, Medical School, Attiko University Hospital, Athens, GRC; 2 Department of Obstetrics and Gynecology, General Hospital of Chania, Chania, GRC; 3 Postgraduate Program "High-Risk Pregnancy", National and Kapodistrian University of Athens, Medical School, Attiko University Hospital, Athens, GRC; 4 Second Department of Obstetrics and Gynecology, National and Kapodistrian University of Athens, Medical School, Aretaieio University Hospital, Athens, GRC

**Keywords:** birth weight, fetal macrosomia, greece, macrosomia, macrosomic, neonatal macrosomia, perinatal medicine, time trends

## Abstract

Objectives: The present study aimed to estimate macrosomia rates in Greece and investigate their temporal trends during the period 1980-2023.

Methods: This nationwide population-based study analyzed official birth registry data obtained from the Hellenic Statistical Authority. The dataset included 4,593,229 live births registered in Greece between 1980 and 2023. For each year, overall macrosomia rates (birth weight ≥4,000 g) and severe macrosomia rates (≥4,500 g) were calculated. Additional analyses were performed separately for grade 1 (4,000-4,499 g), grade 2 (4,500-4,999 g), and grade 3 (≥5,000 g) macrosomia, according to gestational age at delivery (37-39 vs. ≥40 weeks), and among singleton live births. Temporal trends were evaluated using Joinpoint regression analysis, and annual percent change (APC) and average annual percent change (AAPC) values with 95% confidence intervals (95% CI) were calculated.

Results: During 1980-2023, the overall macrosomia rate declined by 63%, from 7.66% in 1980 to a historic low of 2.85% in 2023 (AAPC = −2.3, 95% CI: −2.5 to −2.2). The decrease was moderate during 1980-1992 (APC = −1.5, 95% CI: −2.1 to −0.5) and became particularly pronounced between 1992 and 2001 (APC = −4.4, 95% CI: −7.8 to −3.6) and again during 2007-2010 (APC = −10.2, 95% CI: −12.2 to −6.5), following a period of stabilization between 2001 and 2007. After a modest increase during 2010-2020 (APC = 1.4, 95% CI: 0.8 to 2.9), rates declined again during 2020-2023 (APC = −6.9, 95% CI: −12.1 to −3.1). Severe macrosomia (≥ 4,500 g) decreased by 81%, from 10.17 to a record low of 1.95 per 1,000 live births between 1980 and 2023 (AAPC = −3.9, 95% CI: −4.2 to −3.6). Grade 1 macrosomia (4,000-4,499 g), grade 2 macrosomia (4,500-4,999 g), and grade 3 macrosomia (≥ 5,000 g) declined, with corresponding AAPC values of −2.2 (95% CI: −2.3 to −2.0), −3.4 (95% CI: −3.7 to −3.0), and −6.2 (95% CI: −6.8 to −5.7), respectively. Macrosomia rates decreased more markedly among births at 37-39 gestational weeks (AAPC = −2.8, 95% CI: −3.0 to −2.6) than among births at ≥ 40 weeks (AAPC = −1.0, 95% CI: −1.3 to −0.8). Similar declining trends were observed among singleton live births, with the overall macrosomia rate decreasing from 7.80% to 3.02% and severe macrosomia decreasing from 10.36 to 2.06 per 1,000 singleton live births.

Conclusions: Since 1980, Greece has achieved substantial reductions in macrosomia rates, which reached historically low levels in 2023. The decline was more pronounced for the more severe categories of macrosomia. Continued surveillance and targeted interventions in high-risk populations may further improve fetal growth outcomes in the country.

## Introduction

Macrosomia refers to excessive fetal growth and is typically defined as an absolute birth weight ≥4,000 g, irrespective of gestational age and fetal sex [1]. The term originates from the Greek words makros (μακϱός, large) and soma (σώμα, body) and is used to describe an abnormally large body size. It is believed that the term was introduced into medical terminology in the mid-19th century by the English physician Robley Dunglison [2].

Unlike large for gestational age (LGA), the absolute definition of macrosomia based solely on birth weight does not account for gestational age or fetal sex; however, it offers important methodological advantages, including a fixed cutoff, ease of calculation, and suitability for temporal and international comparisons, as it does not rely on gestational age-specific growth curves or potentially inaccurate gestational age estimation [3,4].

Fetal macrosomia is a significant risk factor for maternal and neonatal morbidity and mortality, as it is associated with numerous complications affecting both the mother and the neonate. Maternal complications include perineal trauma and postpartum hemorrhage, while neonatal complications include shoulder dystocia, brachial plexus injury, fetal distress, hypoglycemia, hyperbilirubinemia, electrolyte imbalances, and admission to the neonatal intensive care unit [2,5,6]. In addition, fetal macrosomia has been linked to an increased risk of long-term adverse outcomes in the offspring, including obesity, metabolic disorders, cardiovascular disease, and cancer [7,8]. Macrosomia is typically classified into grade 1 (4,000-4,499 g), grade 2 (4,500-4,999 g), and grade 3 (≥5,000 g), with the risk of complications increasing markedly with increasing birth weight [6,9,10].

Although various anthropometric and constitutional factors may influence fetal birth weight, the principal risk factors for macrosomia include maternal obesity, excessive gestational weight gain, and gestational diabetes mellitus [1,6,11]. In parallel with the increasing prevalence of these risk factors worldwide, the incidence of macrosomia has risen, particularly in less-developed countries. Considerable variation in macrosomia rates has also been reported between countries [4,12,13]. Given the public health importance of macrosomia, the present study aimed to estimate macrosomia rates in Greece and examine their temporal trends over the period 1980-2023.

## Materials and methods

Study population

This study used publicly available data from the Hellenic Statistical Authority [14], sourced from official birth certificate records. The dataset included all live births registered in Greece from 1980 to 2023, categorized by birth weight (in grams). Across the study period, 4,593,229 live births with complete data were included, accounting for 99.73% of all live births.

Study parameters

For each year, the following indicators were initially calculated for all live births: the overall macrosomia rate (birth weight ≥ 4,000 g) per 100 total live births and the severe macrosomia rate (birth weight ≥ 4,500 g) per 1,000 total live births. Subsequently, the following indicators were also calculated separately for all live births: the grade 1 macrosomia rate (birth weight 4,000-4,499 g) per 100 total live births, the grade 2 macrosomia rate (4,500-4,999 g) per 1,000 total live births, and the grade 3 macrosomia rate (≥ 5,000 g) per 10,000 total live births. Next, macrosomia rates (birth weight ≥ 4,000 g) per 100 live births were calculated separately for live births at 37-39 completed weeks of gestation (37+0 to 39+6 weeks of gestation) and at ≥ 40 completed weeks of gestation. Finally, overall macrosomia rates (birth weight ≥ 4,000 g) and severe macrosomia rates (birth weight ≥ 4,500 g) were calculated for singleton live births as follows: macrosomia rate, the number of singleton live births weighing ≥ 4,000 g per 100 singleton live births; severe macrosomia rate, the number of singleton live births weighing ≥ 4,500 g per 1,000 singleton live births.

Statistical analysis

The macrosomia rates were calculated after entering the data into Microsoft Excel 2010 (Microsoft Corporation, Redmond, Washington). Temporal trends were assessed using the Joinpoint Regression Program, version 6.0.0 (National Cancer Institute, Bethesda, Maryland), which identifies joinpoints corresponding to statistically significant changes in temporal trends using a log-linear model. The selection of the optimal number of joinpoints for the best-fitting model was based on Monte Carlo permutation tests. For each segment between two joinpoints, the annual percent change (APC) and corresponding 95% confidence intervals (CIs) were calculated. Additionally, the average annual percent change (AAPC) was calculated to summarize overall temporal trends across multiple segments. The statistical significance of APC and AAPC estimates was assessed using two-sided t-tests to determine whether the slope differed significantly from zero, with p < 0.05 considered statistically significant. The maximum number of allowed segments was seven, as suggested by the software according to the duration of the study period. In retrospect, this proved sufficient, as no analysis yielded more than six segments (five joinpoints).

## Results

**Figure 1:**
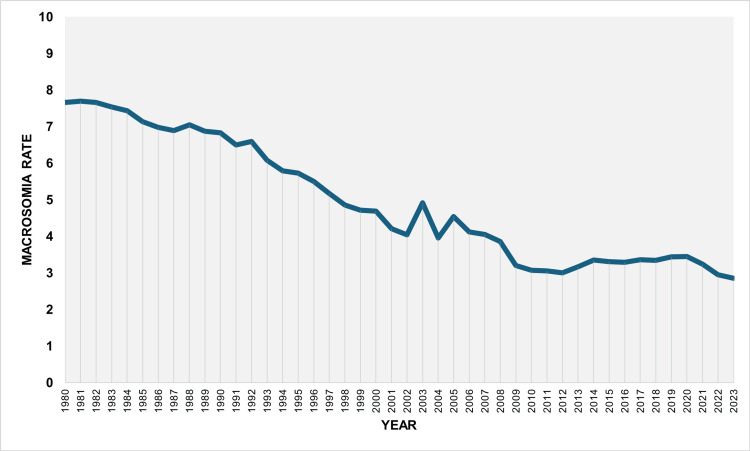
Macrosomia rate (per 100 total live births) in Greece, 1980-2023.

During 1980-2023, the overall macrosomia rate among all live births was 5.10%. The rate decreased from 7.66% in 1980 to a minimum value of 2.85% in 2023, corresponding to a 63% reduction (approximately a 2.7-fold decrease). The highest rate during the study period was observed in 1981 (7.69%). The rate fell below 5% in 1998, remained below 4% from 2008 onward, and dropped below 3% in 2022 and 2023 (Figure 1). 

**Figure 2:**
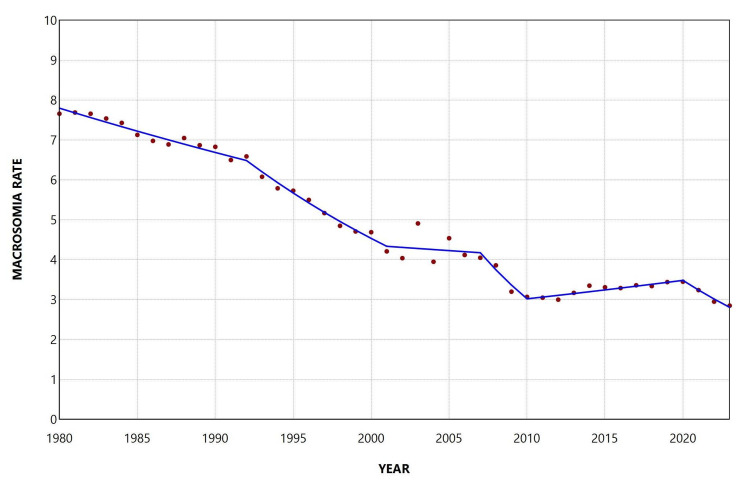
Trends in macrosomia rate (per 100 total live births) in Greece, 1980-2023.

The macrosomia rate decreased overall during the period 1980-2023, with an AAPC of −2.3 (95% CI: −2.5 to −2.2, p < 0.001). The proportion of macrosomic neonates among all births in the country showed a declining trend from 1980 to 2001, with a moderate decrease during 1980-1992 (APC = −1.5, 95% CI: −2.1 to −0.5), followed by a steeper decline during 1992-2001 (APC = −4.4, 95% CI: −7.8 to −3.6). This was followed by a period of stabilization between 2001 and 2007 and then a marked decrease during 2007-2010 (APC = −10.2, 95% CI: −12.2 to −6.5). Interestingly, an increasing trend was observed during 2010-2020, with an annual increase of 1.4% (95% CI: 0.8% to 2.9%), which was subsequently reversed in the most recent period, 2020-2023 (APC = −6.9, 95% CI: −12.1 to −3.1) (Figure 2, Table 1). 

**Table 1:** Trends in macrosomia rate among total live births in Greece, 1980-2023. *Statistically significant.

Segment	Annual percent change	95% confidence interval	P-value
1980-1992	-1.5	-2.1 to -0.5	0.022*
1992-2001	-4.4	-7.8 to -3.6	0.001*
2001-2007	-0.6	-1.9 to 4.0	0.601
2007-2010	-10.2	-12.2 to -6.5	0.002*
2010-2020	1.4	0.8 to 2.9	0.001*
2020-2023	-6.9	-12.1 to -3.1	<0.001*

**Figure 3:**
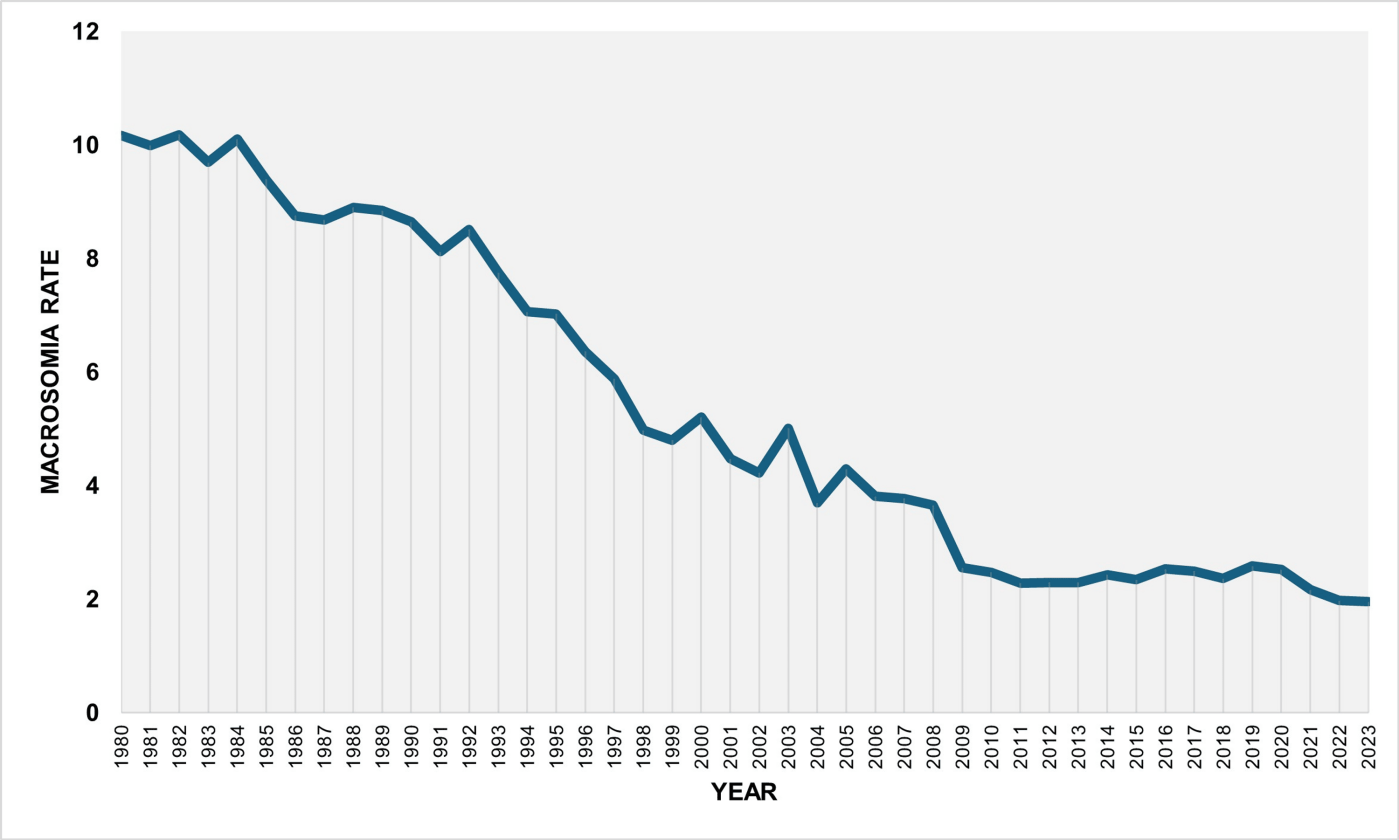
Trends in macrosomia rate ≥ 4,500 g (per 1,000 total live births) in Greece, 1980-2023.

During the period 1980-2023, the overall rate of macrosomia ≥ 4,500 g was 5.69 per 1,000 live births. The rate decreased from 10.17 per 1,000 live births in 1980 to a minimum value of 1.95 per 1,000 live births in 2023, representing a 5.2-fold reduction (81% decrease). The highest rate was observed in 1982 (10.18 per 1,000 live births). The rate remained consistently below 3 per 1,000 live births from 2009 onward and below 2 per 1,000 live births in the two most recent years, 2022 and 2023 (Figure 3). 

**Figure 4:**
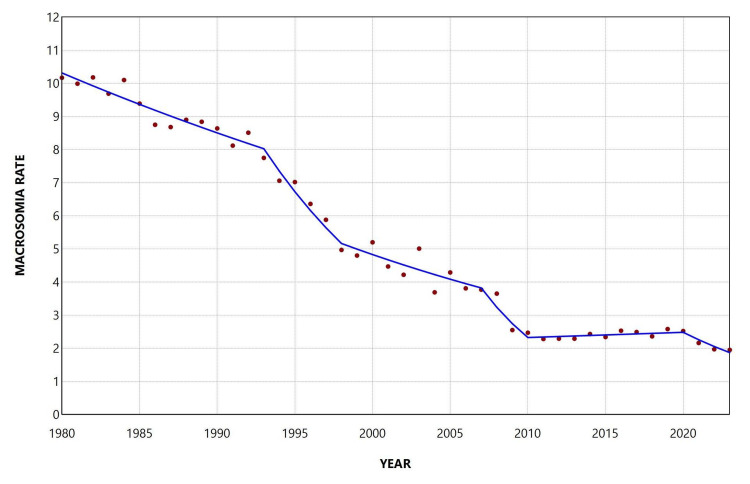
Trends in macrosomia rate ≥ 4,500 g (per 1,000 total live births) in Greece, 1980-2023.

Overall, the rate declined during 1980-2023, with an AAPC of −3.9 (95% CI: −4.2 to −3.6, p < 0.001). In the joinpoint analysis, the decline was statistically significant from 1980 to 2001 (1980-1992: APC = −1.5, 95% CI: −2.1 to −0.5; 1992-2001: APC = −4.4, 95% CI: −7.8 to −3.6), followed by a period of stabilization thereafter, with the exception of two periods of steep decline during 2007-2010 (APC = −10.2, 95% CI: −12.2 to −6.5) and 2020-2023 (APC = −6.9, 95% CI: −12.1 to −3.1) (Figure 4; Table 2). 

**Table 2:** Trends in macrosomia rate ≥ 4,500 g among total live births in Greece, 1980-2023. *Statistically significant.

Segment	Annual percent change	95% confidence interval	P-value
1980-1993	-1.9	-2.7 to -0.8	0.017*
1993-1998	-8.4	-13.0 to -5.4	0.014*
1998-2007	-3.3	-4.6 to 2.1	0.145
2007-2010	-15.3	-18.2 to -8.8	0.006*
2010-2020	0.7	-0.4 to 4.3	0.170
2020-2023	-9.0	-16.3 to -3.1	<0.001

**Figure 5:**
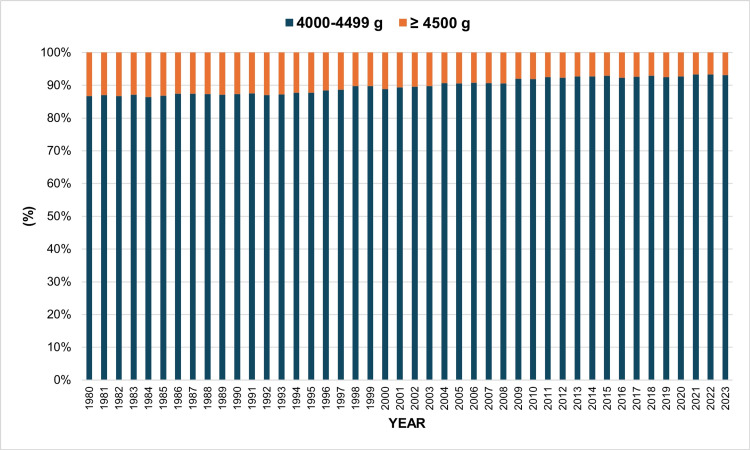
Percentage distribution of macrosomic live births by birth weight group (4,000-4,499 g and ≥4,500 g) in Greece, 1980–2023.

Among all macrosomic live births, the proportion of neonates with severe macrosomia (≥4,500 g) showed a progressive decline, from 13.3% in 1980 to 6.8% in 2023. The highest proportion was observed in 1984 (13.6%), while the lowest percentage was recorded in 2021 and 2022 (6.7%). In contrast, the proportion of macrosomic neonates with a birth weight of 4,000-4,499 g showed an increasing trend, rising from 86.7% in 1980 to 93.2% in 2023 (Figure 5). 

**Figure 6:**
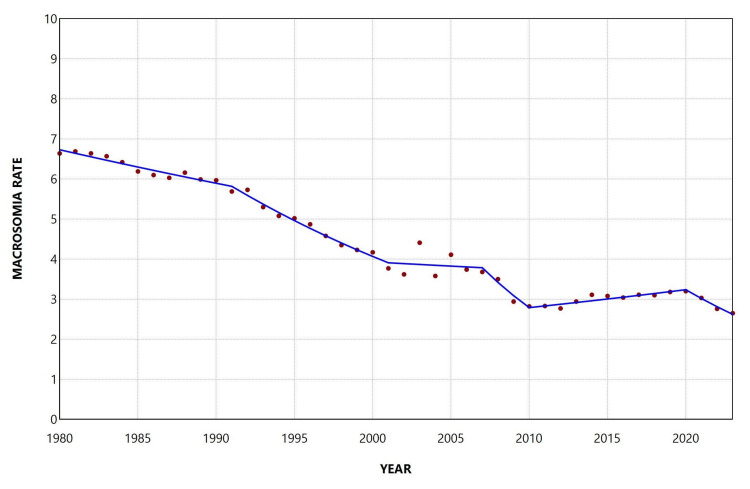
Trends in macrosomia rate 4,000-4,499 g (per 100 total live births) in Greece, 1980-2023

The rate of grade 1 macrosomia (4,000-4,499 g) declined from 6.64% in 1980 to 2.65% in 2023, the lowest value observed during the study period, with an AAPC of −2.2 (95% CI: −2.3 to −2.0, p < 0.001). The peak rate was recorded in 1981 (6.69%). Overall, this represents a 2.5-fold decrease (60% reduction) between 1980 and 2023. The decreasing trend was statistically significant throughout the study period, except for the 2001-2007 segment (Figure 6, Table 3). 

**Table 3:** Trends in macrosomia rate 4,000-4,499 g in Greece, 1980-2023. *Statistically significant.

Segment	Annual percent change	95% confidence interval	P-value
1980-1991	-1.3	-1.9 to -0.0	0.048*
1991-2001	-3.9	-7.1 to -3.2	0.002*
2001-2007	-0.5	-1.9 to 3.9	0.668
2007-2010	-9.7	-11.6 to -6.0	0.002*
2010-2020	1.5	0.9 to 2.9	0.001*
2020-2023	-6.8	-11.9 to -3.0	<0.001*

**Figure 7:**
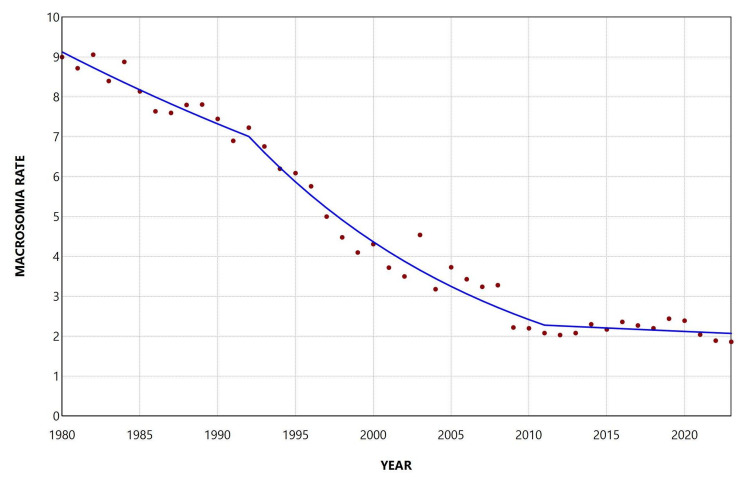
Trends in macrosomia rate 4,500-4,999 g (per 1,000 total live births) in Greece, 1980-2023.

The rate of grade 2 macrosomia (4,500-4,999 g) declined by an average of 3.4% per year (95% CI: −3.7% to −3.0%, p < 0.001). The rate decreased from 9.00 per 1,000 live births in 1980 to a nadir of 1.86 per 1,000 live births in 2023, whereas the peak rate was observed in 1982 (9.06 per 1,000 live births). Overall, this represents a 79% reduction, or a 4.9-fold decrease, between 1980 and 2023. Joinpoint analysis showed a significant decline during 1992-2011 (APC = −5.7, 95% CI: −9.4 to −5.0), while trends during 1980-1992 and 2011-2023 were not statistically significant (Figure 7, Table 4). 

**Table 4:** Trends in macrosomia rate 4,500-4,999 g in Greece, 1980-2023. *Statistically significant.

Segment	Annual percent change	95% confidence interval	P-value
1980-1992	-2.2	-3.6 to 1.1	0.102
1992-2011	-5.7	-9.4 to -5.0	0.001*
2011-2023	-0.8	-2.3 to 2.0	0.401

**Figure 8:**
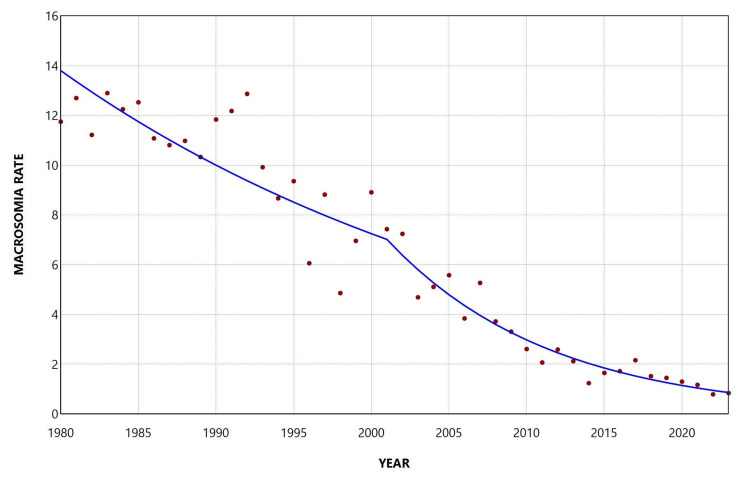
Trends in macrosomia rate ≥ 5,000 g (per 10,000 total live births) in Greece, 1980-2023.

The rate of grade 3 macrosomia (≥ 5,000 g) declined from 11.75 per 10,000 live births in 1980 to 0.84 per 10,000 live births in 2023, representing a 93% reduction (approximately a 14-fold decrease). In 2023, approximately 1 in 11,900 live births weighed ≥ 5,000 g. The peak rate during the study period was observed in 1983 (12.90 per 10,000 live births), while the lowest rate was recorded in 2022 (0.79 per 10,000 live births). Overall, the rate declined continuously over the study period, with an AAPC of −6.2 (95% CI: −6.8 to −5.7, p < 0.001). Joinpoint analysis showed a significant decrease during 1980-2001 (APC = −3.2, 95% CI: −4.4 to −1.4), followed by a steeper decline during 2001-2023 (APC = −9.1, 95% CI: −10.6 to −8.1) (Figure 8, Table 5). 

**Table 5:** Trends in macrosomia rate ≥ 5,000 g in Greece, 1980-2023. *Statistically significant.

Segment	Annual percent change	95% confidence interval	P-value
1980-2001	-3.2	-4.4 to -1.4	0.007*
2001-2023	-9.1	-10.6 to -8.1	<0.001*

**Figure 9:**
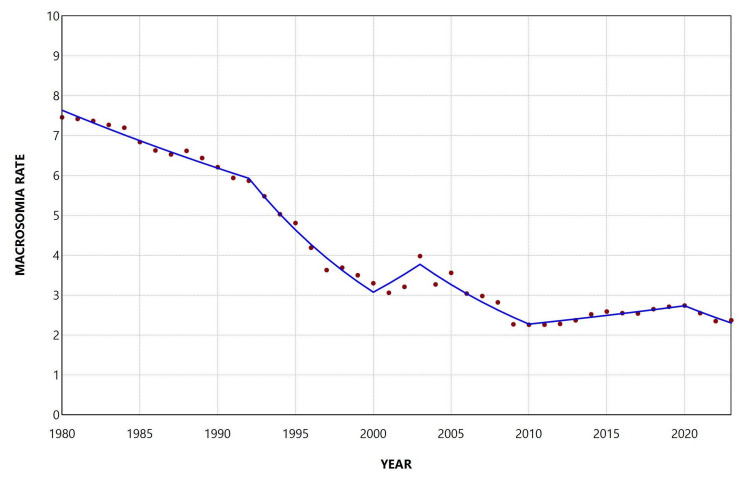
Trends in macrosomia rate at 37-39 weeks of gestation (per 100 live births) in Greece, 1980-2023.

The macrosomia rate at 37-39 weeks of gestation decreased by 68% between 1980 and 2023, corresponding to an approximately 3.1-fold reduction, from a period peak of 7.46% to 2.37% (the period nadir was 2.35% in 2022). Overall, the decline occurred with an AAPC of −2.8 (95% CI: −3.0 to −2.6, p < 0.001). The main reduction in the rate occurred during the first two decades, particularly during the 1990s (1980-1992: APC = −2.1, 95% CI: −2.7 to −1.3; 1992-2000: APC = −7.9, 95% CI: −9.8 to −6.8). After 2000, the trend showed alternating patterns, with increasing trends during 2000-2003 and 2010-2020, and decreasing trends during 2003-2010 and 2020-2023 (Figure 9, Table 6). 

**Table 6:** Trends in macrosomia rate at 37-39 weeks of gestation in Greece, 1980-2023. *Statistically significant.

Segment	Annual percent change	95% confidence interval	P-value
1980-1992	-2.1	-2.7 to -1.3	0.001*
1992-2000	-7.9	-9.8 to -6.8	0.002*
2000-2003	7.1	1.4 to 9.8	0.019*
2003-2010	-7.0	-9.3 to -5.7	0.010*
2010-2020	1.9	1.0 to 3.9	0.010*
2020-2023	-5.6	-11.5 to -1.3	0.011*

**Figure 10:**
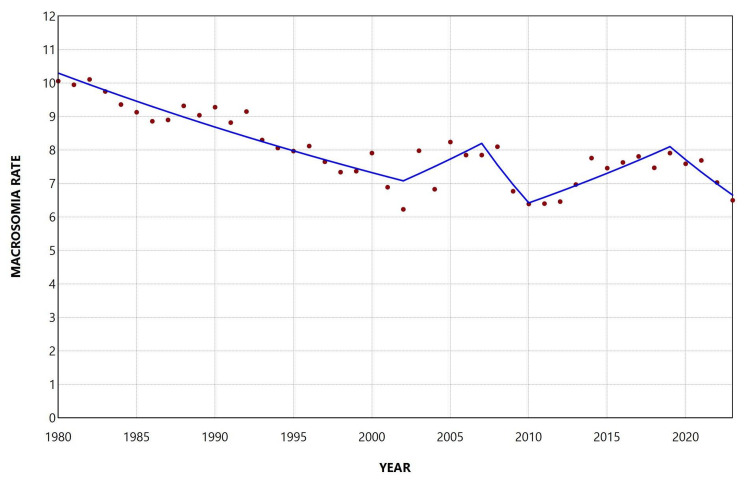
Trends in macrosomia rate at ≥40 weeks of gestation (per 100 live births) in Greece, 1980-2023.

The macrosomia rate at ≥ 40 weeks of gestation decreased by 35%, from 10.06% in 1980 to 6.50% in 2023. Overall, the decline occurred with an AAPC of −1.0 (95% CI: −1.3 to −0.8, p < 0.001). The peak rate was observed in 1982 (10.11%). The main reduction in the rate occurred during the first two decades, when the rate declined to the lowest value of the period in 2002 (6.23%) (1980-2002: APC = −1.7, 95% CI: −2.1 to −1.4). Since then, the rate has shown increasing trends, with the exception of two periods of decline during 2007-2010 and 2019-2023. From 2002 to 2023, the overall trend was not statistically significant (AAPC = −0.3, 95% CI: −0.9 to 0.1) (Figure 10, Table 7). 

**Table 7:** Trends in macrosomia rate at ≥ 40 weeks of gestation in Greece, 1980-2023. *Statistically significant.

Segment	Annual percent change	95% confidence interval	P-value
1980-2002	-1.7	-2.1 to -1.4	0.009*
2002-2007	3.0	0.4 to 7.7	0.033*
2007-2010	-7.8	-10.8 to -2.5	0.008*
2010-2019	2.6	1.6 to 7.3	0.007*
2019-2023	-4.8	-11.0 to -1.2	0.008*

**Figure 11:**
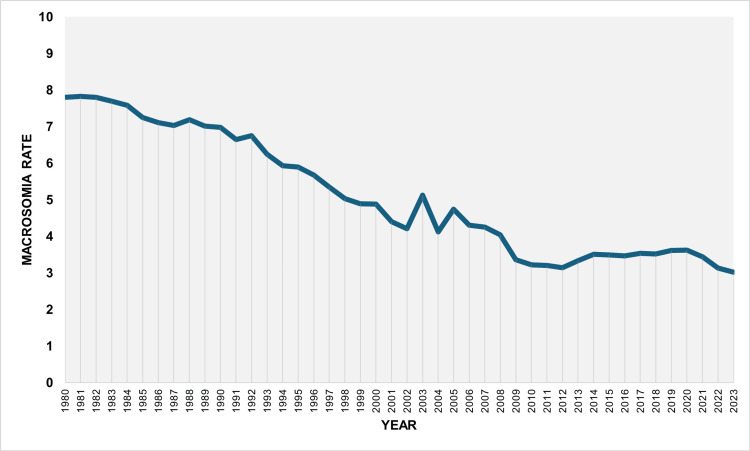
Macrosomia rate (per 100 single live births) in Greece, 1980-2023.

During 1980-2023, the macrosomia rate among singleton live births was 5.29%. The rate declined from 7.80% in 1980 to a minimum value of 3.02% in 2023, corresponding to a 61% reduction (approximately a 2.76-fold decrease). The highest rate during the study period was observed in 1981 (7.82%). The macrosomia rate among singleton births remained below 4% from 2009 onward (Figure 11). 

**Figure 12:**
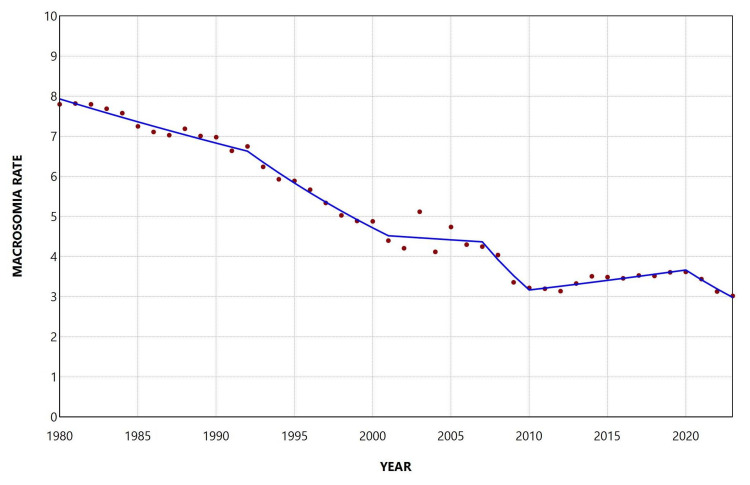
Trends in macrosomia rate (per 100 single live births) in Greece, 1980-2023.

Overall, the macrosomia rate among singletons decreased during 1980-2023, with an AAPC of −2.2 (95% CI: −2.4 to −2.1, p < 0.001). The trend pattern was similar to that observed for total live births, with declining trends from 1980 to 2001, followed by a period of stabilization between 2001 and 2007 and a marked decrease during 2007-2010. In more recent years, an increasing trend was observed during 2010-2020, which was subsequently reversed during the most recent period, 2020-2023 (Figure 12, Table 8). 

**Table 8:** Trends in macrosomia rate among single live births in Greece, 1980-2023. *Statistically significant.

Segment	Annual percent change	95% confidence interval	P-value
1980-1992	-1.5	-2.0 to -0.4	0.029*
1992-2001	-4.2	-7.6 to -3.4	0.002*
2001-2007	-0.6	-1.9 to 4.0	0.643
2007-2010	-10.2	-12.1 to -6.4	0.002*
2010-2020	1.5	0.8 to 3.0	0.002*
2020-2023	-6.7	-11.8 to -2.7	0.001*

**Figure 13:**
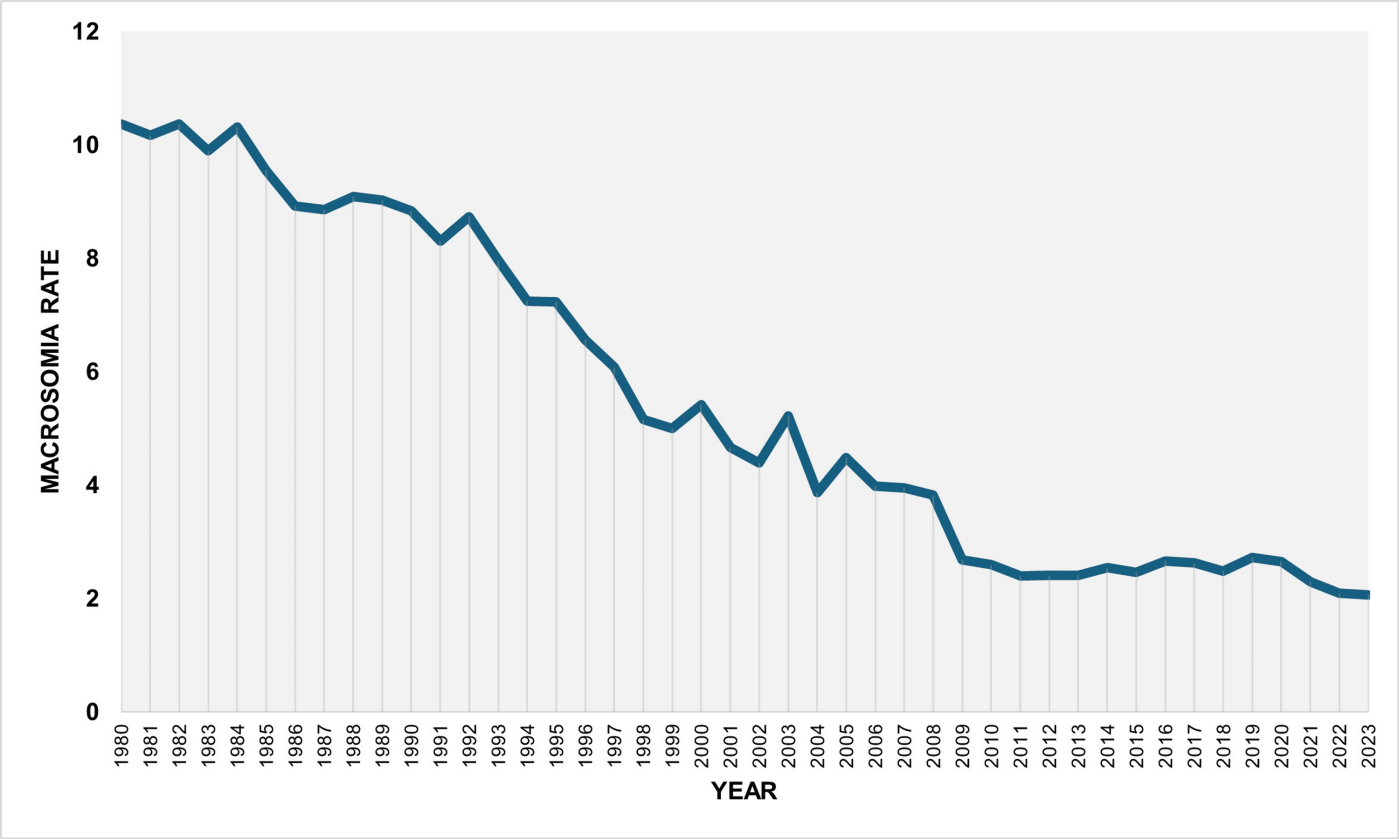
Macrosomia rate ≥4,500 g (per 1,000 single live births) in Greece, 1980-2023.

The macrosomia rate ≥ 4,500 g among singleton live births decreased from 10.36 per 1,000 live births in 1980 (period peak) to 2.06 per 1,000 live births (period low) in 2023, representing an 80% reduction (approximately a fivefold decrease). The rate remained below 3 per 1,000 live births from 2009 onward (Figure 13). 

**Figure 14:**
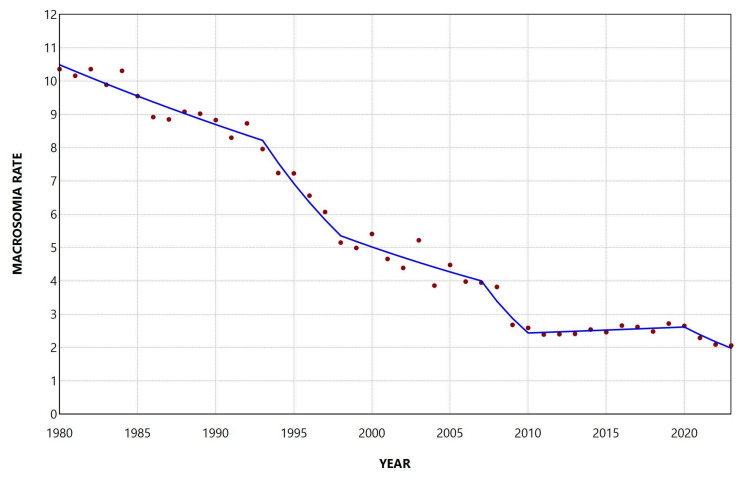
Trends in macrosomia rate ≥4,500 g (per 1,000 single live births) in Greece, 1980-2023.

Between 1980 and 2023, the macrosomia rate (≥ 4,500 g) among singletons decreased, with an AAPC of −3.8 (95% CI: −4.1 to −3.5, p < 0.001). The rate declined from 1980 to 1998. Subsequently, from 1998 to 2020, the rate appeared to stabilize, with a sharp decline during 2007-2010. Finally, a statistically significant decline was observed during 2020-2023 (Figure 14, Table 9). 

**Table 9:** Trends in macrosomia rate (≥ 4,500 g) among single live births in Greece, 1980-2023. *Statistically significant.

Segment	Annual percent change	95% confidence interval	P-value
1980-1993	-1.9	-2.6 to -0.7	0.020*
1993-1998	-8.2	-12.7 to -5.1	0.018*
1998-2007	-3.2	-4.5 to 2.2	0.152
2007-2010	-15.2	-18.2 to -8.9	0.006*
2010-2020	0.7	-0.3 to 4.3	0.140
2020-2023	-8.9	-16.0 to -3.0	<0.001*

## Discussion

In this nationwide analysis of live births in Greece during 1980-2023, we observed a marked decline in the prevalence of macrosomia across all birth-weight categories. The reduction in macrosomia rates was particularly pronounced during the 1990s and again during 2007-2010. Notably, the lowest rates ever recorded in Greece were observed in 2023, reaching 2.85% for the overall macrosomia rate and 1.95 per 1,000 live births for severe macrosomia (≥4,500 g).

The reduction in macrosomia rates between 1980 and 2023 was more pronounced for the more severe categories of macrosomia. The overall macrosomia rate decreased by 63%, severe macrosomia (≥ 4,500 g) declined by 81%, and the reduction was even greater for grade 3 macrosomia (≥ 5,000 g), which decreased by 93%. Likewise, the rate of decline became progressively steeper with increasing birth-weight category, with AAPC values of −2.2%, −3.4%, and −6.2% for grade 1 (4,000-4,499 g), grade 2 (4,500-4,999 g), and grade 3 (≥5,000 g) macrosomia, respectively.

Over time, the proportion of severe macrosomia among all macrosomic births progressively declined. While neonates weighing 4,500-4,999 g and ≥5,000 g became increasingly rare, grade 1 macrosomia (4,000-4,499 g) accounted for a progressively larger proportion of all macrosomic births. This finding is clinically important because the risk of neonatal complications increases substantially with increasing macrosomia severity [15]. The observed trends likely reflect improvements in antenatal surveillance and obstetric management, including earlier diagnosis and treatment of gestational diabetes, closer fetal growth monitoring, and increased use of labor induction in pregnancies suspected of excessive fetal growth [16]. Nevertheless, antenatal diagnosis of fetal macrosomia remains challenging and relatively imprecise [17].

Subsequently, macrosomia rates in Greece were analyzed according to gestational age at delivery. The decline in macrosomia was substantially greater among births at 37-39 gestational weeks than among births at ≥40 weeks. Notably, the entire improvement in macrosomia rates among pregnancies reaching ≥40 weeks was achieved by 2002, whereas thereafter the downward trend was no longer statistically significant. The persistence of relatively high macrosomia rates among pregnancies ≥40 weeks may suggest the existence of a subgroup of women with insufficient antenatal surveillance during late pregnancy, resulting in continued excessive fetal growth toward term.

Finally, the analysis was extended to investigate macrosomia rates exclusively among singleton pregnancies. The declining trends observed were similar to those identified for total live births. The overall and severe (≥4,500 g) macrosomia rates among singleton births decreased by 61% and 80%, respectively, reaching historically low levels in 2023 of 3.02% and 2.06 per 1,000 live births, respectively. The calculation of macrosomia rates specifically among singleton births is biologically meaningful, as singleton pregnancies are essentially the only pregnancies at substantial risk for macrosomia. Conversely, the assessment of macrosomia rates among all live births provides important public health information by reflecting the overall population burden of macrosomia at the national level.

During the period 1980-2023, the steepest declines in macrosomia rates in Greece occurred during two relatively short time intervals: (1) 2007-2010, corresponding to the initial phase of the severe economic crisis in Greece, and (2) 2020-2023, a period characterized by marked socioeconomic turbulence related to the COVID-19 pandemic [18,19]. Both periods were also accompanied by reductions in births to immigrant mothers. Previous studies from Southern Europe have reported a higher prevalence of macrosomic neonates among immigrant women [20]. Notably, between 2020 and 2023, the proportion of live births in Greece to mothers of foreign nationality declined by 28%, from 14.8% to 10.7% [14].

The remarkable decline in macrosomia rates has resulted in Greece having among the lowest macrosomia rates reported in developed countries. In 2023, the macrosomia rate in Greece was approximately 2.5-fold lower than that reported in the United States (2.85% vs. 7.10%) [21]. Although macrosomia rates in the United States have also declined in recent years, substantial differences persist across racial and ethnic groups [22,23]. Similarly, considerably higher macrosomia rates have been reported in China, reaching 5.9% in 2021 [24]. Even in low- and middle-income countries, the overall macrosomia rate has been estimated at approximately 7%, whereas lower prevalences of macrosomia have been described among South Asian populations [25]. High prevalences, albeit with considerable intercountry variation, have also been documented across several Asian countries [26]. Furthermore, according to the European Perinatal Health Report based on 2019 data, Greece, together with Cyprus, had the lowest rate of severe macrosomia (≥ 4,500 g) among European countries (0.2%), compared with a median value of 1.1% across Europe [27]. Countries in Southern Europe appeared to have relatively low macrosomia rates. These international differences likely reflect variations in anthropometric characteristics, cultural and dietary habits, lifestyle factors, as well as differences in obstetric clinical practice.

The substantial improvement in macrosomia rates in Greece represents an important public health achievement, given the serious short- and long-term consequences of excessive fetal growth for neonatal health and later adult life. This improvement was particularly pronounced for severe forms of macrosomia. The macrosomia rate (≥ 4,500 g) in Greece was fourfold lower than that reported in the United States in 2022 (0.21% vs. 0.84%, for singletons) [22]. Moreover, the rate of macrosomia ≥5,000 g declined to nearly negligible levels (<1 per 10,000 live births). Compared with the United States in 2022, the grade 3 macrosomia rate (≥5,000 g) in Greece was 20-fold lower (0.8 vs. 16 per 10,000 singleton live births) [22]. Further investigation is needed to clarify the maternal factors, particularly metabolic determinants such as obesity prevalence, gestational weight gain, and gestational diabetes, that may have contributed to the declining trends observed. The very low macrosomia rates in Greece are also likely related to the exceptionally high rates of prematurity and, especially, early-term births reported in the country [28,29]. Conversely, Greece has the highest low birth weight rates reported in Europe and other Western countries [30].

The present study has several important strengths. First, the study was based on a nationwide birth registry that included more than 4.5 million live births over a 44-year period, with nearly complete national coverage, very high data completeness, and official population-based birth certificate data, ensuring a high level of reliability. In addition, the use of a fixed birth-weight definition of macrosomia facilitated reliable long-term and international comparisons independent of changes in gestational age estimation methods or fetal growth standards. Another major strength was the application of Joinpoint regression analysis, which enabled the identification of distinct time periods during which temporal trends changed significantly. Furthermore, the study separately analyzed different severity categories of macrosomia, allowing a more detailed assessment of changes across the birth-weight spectrum. Additional analyses were also performed according to gestational age at delivery and separately among all live births and singleton births, providing a more comprehensive evaluation of macrosomia trends in the Greek population.

Nevertheless, certain limitations should be acknowledged. The study relied on aggregated national registry data and therefore lacked information on important maternal and pregnancy-related variables, including fetal sex, maternal body mass index, gestational diabetes, parity, gestational weight gain, and socioeconomic status. The absence of individual-level data precluded adjustment for potential confounding factors. Finally, temporal changes in obstetric practices, maternal characteristics, and population demographics may also have influenced the observed trends.

Macrosomia rates in Greece improved markedly between 1980 and 2023, with the most pronounced reductions observed in severe forms of macrosomia. However, these favorable trends may have been achieved, at least in part, at the cost of the high incidence of preterm and early-term deliveries in the country. Continued surveillance of birth weight trends and preventive strategies targeting maternal obesity and gestational diabetes remain important public health priorities. Nevertheless, significant challenges persist, including the stabilization of macrosomia rates among pregnancies reaching ≥40 gestational weeks during the last two decades, the continued presence of high-risk groups such as immigrant women and women with obesity, as well as the increasing use of frozen embryo transfer in IVF pregnancies, which has been associated with an elevated risk of large-for-gestational-age infants and macrosomia [7].

## Conclusions

Since 1980, Greece has achieved remarkable progress in reducing macrosomia rates, which reached historically low levels in 2023 and are now among the lowest reported in Western and developed countries. The decline was particularly pronounced for the more severe categories of macrosomia. However, the limited improvement in macrosomia rates among pregnancies reaching 40 gestational weeks during the last two decades may indicate gaps in late-pregnancy antenatal surveillance. Continued monitoring of macrosomia trends and targeted interventions in high-risk populations remain important for optimizing fetal growth outcomes in the country.
